# OLMSTED SYNDROME

**DOI:** 10.4103/0019-5154.41657

**Published:** 2008

**Authors:** Pramod Kumar, P K Sharma, H K Kar

**Affiliations:** *From Departments of Dermatology and STD, Dr. Ram Manohar Lohia Hospital, New Delhi, India*

**Keywords:** *Genodermatosis*, *hypotrichosis*, *Olmsted syndrome*, *onychodystrophy*, *palmoplantar keratoderma*

## Abstract

Olmsted syndrome is a rare disorder characterized by the combination of periorificial, keratotic plaques and bilateral palmoplantar keratoderma. New associated features are being reported. Olmsted syndrome is particularly rare in a female patient, and we report such a case in a six year-old Indian girl, who presented with keratoderma of her soles since birth and on her palms since the age of two years along with perioral and perinasal hyperkeratosis. She had sparse, light brown, thin hair. Although the psychomotor development of the child was normal until 18 months of age, the keratoderma plaques had restricted the child's mobility after that stage.

## Introduction

Olmsted syndrome (congenital palmoplantar and perioral keratoderma) is a very rare disorder, named after H. C. Olmsted, whose original description of the disorder included a combination of bilateral, mutilating, palmoplantar keratoderma and periorificial hyperkeratotic plaques with flexion deformities of the digits, leading to constriction or spontaneous amputation.^1^ Bilateral palmoplantar keratoderma and periorificial hyperkeratotic plaques are the hallmarks for diagnosis of this condition.^2^

Many features have been subsequently associated with this syndrome and new features continue to be reported. Five cases have now been reported^3^ but only one has been observed in a female so far.^4^ Nevertheless, Olmsted syndrome still remains a rarely reported condition. We report here the case of a six year-old female child, who had bilaterally symmetrical, palmoplantar keratoderma, perioral and perinasal hyperkeratosis, onychodystrophy and hypotrichosis.

## Case History

A six year-old female child presented with excessively thickened skin over her palms and soles. The child was born of a normal vaginal delivery with redness on both her soles; after a few months, the skin over the soles got thickened and started peeling off. Similar thickening began on the palms at the age of two years and warty spots on the sides of the mouth and nose were also noticed. Later, the parents noted that the child's hair was short, not dense and was very slow to grow unlike the children of her age. Her developmental milestones were however, appropriate for her age. The ugly keratoderma had restricted her mobility; hence, the child could not mingle with her peers. There was no history of hyperhidrosis, consanguinity or any skin disease in the family.

**Fig. 1:**
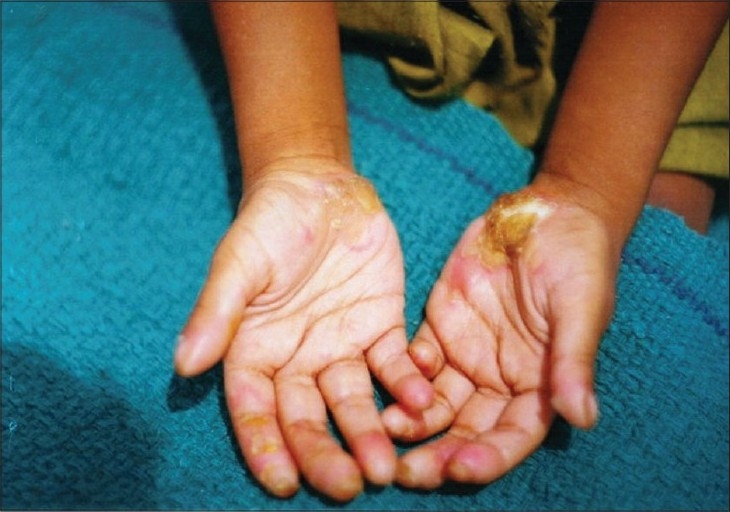
Keratotic plaques on palms

**Fig. 2:**
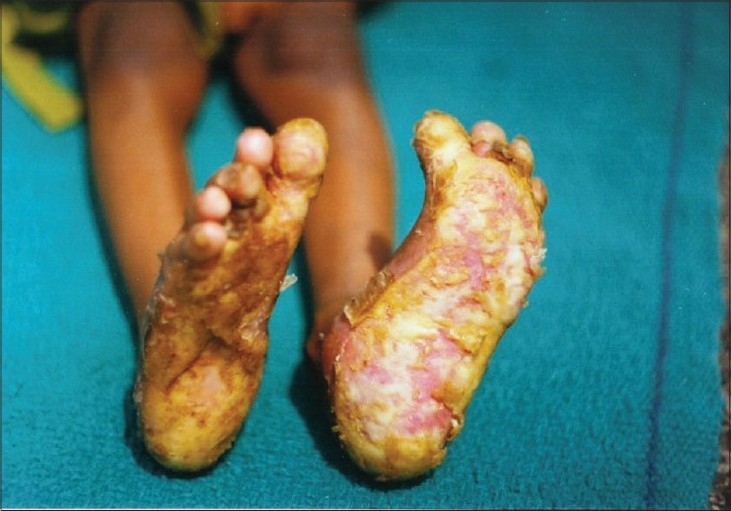
Keratotic sandals

**Fig. 3:**
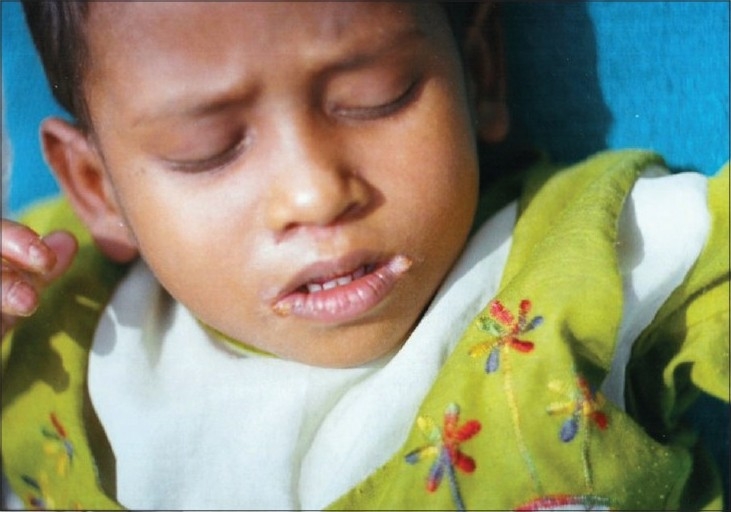
Keratotic plaques on the angles of the mouth and the nares

Examination revealed an active girl with apparently normal intelligence. Her physical parameters were within normal limits for her age. Cutaneous examination revealed thick, hyperkeratotic plaques with underlying erythema on the thenar eminence of both palms and fingers (Fig. 1). The keratotic plaques were hard, well defined, 3 cm × 4 cm in size, thick in places and thin in others and extended to the dorsum of the distal phalanges. The lesions on the soles had formed ‘Keratotic Sandals’ (Fig. 2) with underlying erythema and thick adherent scales. Keratoderma on the soles had extended to involve the dorsum of the feet along the borders. There were some flexion deformities on both soles. Verrucous plaques were seen on the angles of the mouth and around the nares (Fig. 3). Nails of both hands corresponding to skin involvement on the fingers were lusterless and ridged. Similarly, all toenails were lusterless and rough. The nails on last two toes of the right foot were spared, as was the skin. Hair over the scalp, eyebrows and eyelashes was thin, sparse and light brown in color. Oral, genital and ocular mucosa, joints and teeth were found to be normal.

Routine hemogram, peripheral smear, liver and renal function tests, urine and stool examination did not reveal any abnormality. Blood for venereal diseases research laboratory test was non-reactive. Potassium hydroxide mount of the skin scrapings did not show the presence of any fungus. Serum zinc estimation was not available.

Histopathology examination of the lesions showed thick layer of keratosis, parakeratosis, thickened granular cell layer, acanthosis and irregular elongation of rete ridges. Sparse perivascular mononuclear infiltrate was visible in the papillary dermis. A few sweat ducts were also seen. Hair appeared to have less pigment and hair shafts were thin when observed under a light microscope.

Therapy with oral zinc did not improve the condition at all. Salicylic acid with Clobetasol propionate combination in ointment base did not affect the tough scales. Paring was done and a Urea and Lactic acid combination used, which resulted in some relief.

## Discussion

Olmsted described a five year-old male who developed sharply marginated, palmoplantar keratoderma during his first year of life. The thick keratoderma led to flexion deformities and autoamputation of the digits. Periorificial, sharply marginated, hyperkeratotic plaques were also present.^1^

Reports of new cases have lengthened the list of cutaneous and systemic features that may be seen in this syndrome. However, it is widely accepted that two major findings that are prerequisites for diagnosis of this syndrome are the symmetrical involvement of the palms and soles with keratoderma, and symmetrical hyperkeratotic plaques in the periorificial areas.^2^

Our patient had bilaterally symmetrical palmoplantar keratoderma, symmetrical perioral and perinasal keratotic plaques, onychodystrophy and hypotrichosis. Leukokeratosis, pseudoainhum, joint laxity and dental abnormalities were absent. Other features reported occasionally in this syndrome are hyperkeratotic follicular lesions, linear hyperkeratosis of extensor surfaces on the extremities, hyperhidrosis of the palms and soles, anhidrosis, leukokeratosis, chronic blepharitis, absent premolars, hearing loss, corneal epithelial dysplasia and corneal opacities.^2^

Palmoplantar keratoderma and perioral and perinasal keratotic plaques were diagnostic of Olmsted syndrome in our patient, thus excluding other syndromes of keratodermas including Mal de meleda, Vohwinkel syndrome and Pachyonychia congenita. Failure to respond to oral Zinc therapy ruled out the possibility of acrodermatitis enteropathica.

This syndrome seems to be of sporadic occurrence^4^ although a familial case possibly due to autosomal dominant transmission^5^ and another case with X-linked dominant inheritance in two monozygotic male twins have been reported.^6^ Kress *et al.* found a defect in the expression of mature epidermal keratins (types 1 and 10) and persistence of acidic keratins (types 5 and 14) in the involved epidermis.^7^ The majority of the published cases have been males; it has been rarely reported in females.^8^

The disease has a slow but progressive course. The keratoderma becomes extremely thick and may interfere with normal walking. Fissuring around the toes that heals with a constricting band of tissue, leading to autoamputation of the digits, has been reported.^4^ The periorificial lesions may or may not improve with age.

There is no satisfactory treatment for this condition. Topical treatments offer only symptomatic relief of pain and fissures by reducing the thickness of the keratotic palmoplantar skin lesions. Various topical agents like salicylic acid, urea, boric acid, corticosteroids, shale oil, other emollients, retinoic acid, antimicrobials, wet dressings and prolonged soaking of the affected parts in water, have been tried with varying success. Systemic treatment with antihistamines, vitamins E and A, antimicrobials, corticosteroids have also been used anecdotally with no consistent benefits.^2^
